# Application of Fourier Transform Near-Infrared Spectroscopy and Chemometrics for Quantitative Analysis of Milk of Lime (MOL) Used in the Sugar Industry

**DOI:** 10.3390/molecules30112308

**Published:** 2025-05-24

**Authors:** Radosław Michał Gruska, Alina Kunicka-Styczyńska, Magdalena Molska

**Affiliations:** Department of Sugar Industry and Food Safety Management, Faculty of Biotechnology and Food Science, Lodz University of Technology, Wólczańska Str. 171/173, 90-530 Lodz, Poland; alina.kunicka@p.lodz.pl (A.K.-S.); magdalena.molska@p.lodz.pl (M.M.)

**Keywords:** FT-NIR spectroscopy, chemometrics, milk of lime, calcium oxide, process control, green analytical chemistry

## Abstract

Milk of lime (MOL), a suspension of calcium oxide and calcium hydroxide, is vital in the purification of sugar beet and cane juices. This study evaluates the application of Fourier Transform Near-Infrared (FT-NIR) spectroscopy combined with chemometric models—Partial Least Squares (PLS) and Principal Component Regression (PCR)—for rapid, non-destructive assessment of key MOL parameters: density, total lime content, calcium oxide availability, and sucrose content. Ninety samples were analyzed using both wet chemistry and FT-NIR. The predictive performance was assessed using the coefficient of determination (R^2^). High predictive accuracy was observed for density (PLS: R^2^ = 0.8274; PCR: R^2^ = 0.8795) and calcium oxide availability (PLS: R^2^ = 0.9035; PCR: R^2^ = 0.9115). Total lime content showed moderate accuracy (PLS: R^2^ = 0.7748; PCR: R^2^ = 0.7983), while sucrose content exhibited low predictive power (PLS: R^2^ = 0.2312; PCR: R^2^ = 0.3747). The weak performance was noted for %CaO (PLS: R^2^ = 0.4893; PCR: R^2^ = 0.2409), likely due to spectral overlap and matrix complexity. Despite these challenges, FT-NIR remains a viable, reagent-free method for monitoring MOL, with the potential to enhance process control in the sugar industry. Future work should focus on refining calibration strategies and addressing spectral interferences to improve predictive accuracy for complex matrices.

## 1. Introduction

The sugar industry, a cornerstone of the global food production system, relies on efficient and effective purification processes to produce high-quality sugar from sugar beets and sugarcane. One of the critical components in this purification process is the milk of lime (MOL), a suspension of calcium hydroxide in water. Milk of lime is an integral part of the sugar purification process due to its ability to neutralize acids and facilitate the removal of impurities from raw sugar juice by precipitation. The proper clarification is essential for producing high-purity sugar for consumption and industrial use [1,2,3,4,5,6,7,8].

However, the preparation, handling, and application of MOL is not without its challenges. Variability in lime quality, improper mixing, and storage conditions can lead to inconsistencies that compromise the purification process [4,6,8]. Precise dosing and uniform distribution are critical to avoid the issues of over-liming or under-liming. Over-liming can lead to scaling and fouling of equipment, while under-liming can result in incomplete purification [1,8]. Advancements in lime treatment technologies and improved handling practices are essential to address these challenges. Research indicates that optimizing lime slaking processes and employing advanced monitoring systems can significantly enhance the efficiency of milk of lime usage in sugar production [5,9,10,11]. Regrettably, the optimal dosing of milk of lime necessitates a comprehensive understanding of its physicochemical composition. Physicochemical analyses of MOL are based on time-consuming methods such as titration, neutralization, weighing, material balances, atomic absorption spectrometry (AAS), or X-ray fluorescence (XRF) [12,13,14]. Reliable data on density or the content of other constituents is important for precise dosing and effective utilization in various industrial processes. One of the analytical techniques that provides rapid and precise collecting of information about the examined material is near-infrared spectroscopy, particularly Fourier Transform Near-Infrared Spectroscopy (FT-NIR). FT-NIR spectroscopy, operating in the spectral range of ν = 12,500–4000 cm^−1^, has become a widely recognized analytical technique for rapid, non-destructive quantitative analysis of chemical compositions in various matrices [15,16]. Different compounds can be analyzed both quantitatively and qualitatively by FT-NIR spectroscopy due to their characteristic absorption/emission in the IR spectral region [17]. Simultaneous qualitative identification and quantitative determination of many chemical compounds make FT-NIR spectroscopy suitable for used in control laboratories of varied industries: food, pharmaceutical, chemical, wood, feed, and others [18,19,20,21,22].

There is still scarce research concerning the application of Fourier Transform Near-Infrared (FT-NIR) spectroscopy for the analysis of milk of lime. In sugar production technology, milk of lime is formulated in water solutions of sucrose. This sweet process water is generated during the desweetening of the sludge that remains after raw juice purification. In this context, four parameters of milk of lime are critical: density, total lime content, calcium oxide availability, and sucrose content [23,24]. The total lime content in milk of lime refers to the combined amount of all forms of calcium oxide (CaO) and calcium hydroxide (Ca(OH)_2_) present. It includes both dissolved and undissolved components and serves as a critical indicator of the solution’s reactivity and quality. In turn, calcium oxide availability refers to the portion of calcium oxide (CaO) that is capable of reacting, which may include both the free lime (reacted CaO) and the CaO that has already undergone partial hydration but remains reactive. It accounts for all the calcium oxide that can possibly engage in additional chemical processes, whether in its free or partially hydrated phase. The determination of the first three parameters is essential for the accurate dosing of milk of lime, whereas the sucrose level estimation is crucial for its inclusion in the calculation overall sugar production balance. The literature data demonstrate that FT-NIR spectroscopy can be used not only, as is commonly accepted, for the quantitative determination of organic compounds, e.g., sucrose [25,26,27], but also for the analysis of inorganic materials, e.g., milk of lime. The research is mainly focused on the calcium assessment by FT-NIR spectroscopy in soil analysis [28,29] or the mining industry [30,31,32,33]. Quantitative determination of calcium hydroxide has been investigated by Saeki et al. [34] and Biernacki et al. [35]. Near-infrared spectroscopy (NIR) was also applied to examine the hydration reaction of cement, revealing a notable peak at 7082 cm^−1^ associated with the first overtone of the O-H stretching in calcium hydroxide, as supported by existing studies [36,37,38,39].

The aim of this study was to assess the potential of FT-NIR spectroscopy, in combination with chemometric methods (PLS and PCR), for the rapid and non-destructive determination of density, calcium oxide content, and sucrose concentration, key physicochemical parameters of milk of lime simulating the ones produced under industrial conditions using sweet process water in beet and cane sugar factories.

## 2. Results and Discussion

### 2.1. Physical and Chemical Properties of Milk of Lime (MOL)

Milk of lime was prepared as it was described in Section 3.1. Sucrose solution concentrations were also proposed in the same section. The physicochemical parameters of milk of lime are presented in Table 1.

Studied milk of lime samples, density increased from 1.145 to 1.249 g/cm^3^, with the rise attributable to increasing amounts of added quicklime (CaO) and sucrose. The average density was 1.197 g/cm^3^, which aligns with typical values observed in the sugar industry for MOL used in both beet and cane sugar processing (1.18–1.20 g/cm^3^) [13,40].

The total lime content varied only with the increasing amount of quicklime (CaO) applied. For a quicklime-to-water ratio of 1:6, the total amount of lime averaged 17.76 g CaO/100 cm^3^ and increased to an average of 23.37 g CaO/100 cm^3^ with the quicklime-to-water ratio of 1:4. According to the literature and technical data, the milk of lime with density between 1.14 and 1.25 g/cm^3^ is characterized by total lime in the range of 17–32 g CaO/100 cm^3^ [41].

Calcium oxide availability is accounted for as calcium forms capable of reacting with other components in solutions used in the sugar industry for sugar beet raw juice purification. Calcium oxide availability in the total calcium is the percentage of active calcium in the milk of lime. The analysis of solubility parameters of quicklime in water and sucrose solutions allows for the formulation of two key observations:

1. Increasing the amount of quicklime in pure water leads to a corresponding increase in available calcium oxide content—from 14.53 g CaO/100 cm^3^ at a 1:6 quicklime-to-water ratio to 17.81 g CaO/100 cm^3^ at a 1:4 ratio. Simultaneously, a decrease in the share of calcium oxide available in the total lime content is observed, amounting to 81.67% for the 1:4 ratio and 75.76% for the 1:6 ratio.
2. Sucrose in milk of lime at concentrations exceeding 2–3% *w*/*w* leads to an increase in the available calcium oxide content compared to MOL prepared with pure water. At the highest tested sucrose concentration (5% *w*/*w*), this increase ranged from 4.86 percentage points (quicklime-to-water ratio 1:6) to 5.54 percentage points (1:4 ratio) relative to the corresponding milk of lime samples prepared with pure water.

The observed increase in calcium oxide availability with the increased amount of quicklime added to pure water, accompanying the decrease in the proportion of calcium oxide available in total lime, can be explained by changes in lime reactivity. This reactivity is largely influenced by the specific surface area (SSA) of the quicklime, which includes both the external particle surfaces and the internal pore surfaces formed due to CO_2_ release during carbonate mineral calcination [42]. It is well established that a higher SSA leads to a rapid temperature rise during lime slaking, which may accelerate the hydration reaction and increase the amount of available CaO [14,43]. Consequently, adding a greater amount of quicklime results in a higher calcium oxide availability value; however, its proportion in the total lime decreases, suggesting that not all of the added lime mass participates in the reaction with water under the given experimental conditions.

**Figure 1:**
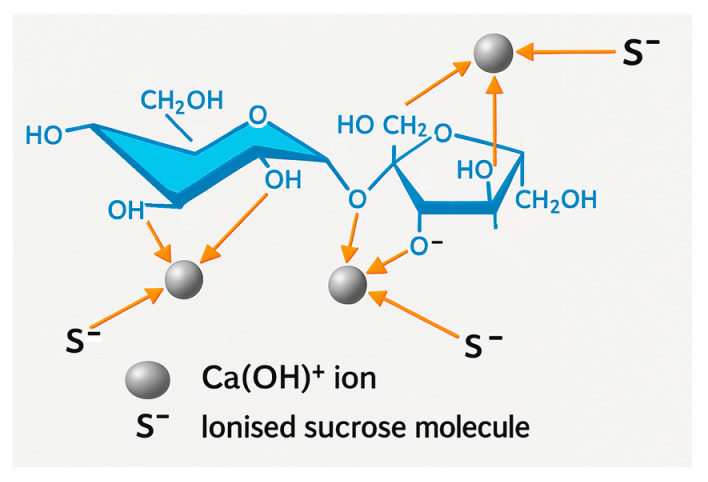
Possible interactions of calcium hydroxy ions with sucrose molecules via coordination bonds. Yellow arrows represent coordination bonds. Drawing prepared on the basis of [48].

The increase in calcium oxide availability in milk of lime and in total lime with the addition of sucrose can be explained by the enhancement of the solubility of quicklime, which is in agreement with literature data [44,45,46]. Calcium ions form complexes with carbohydrates such as sucrose, and in alkaline settings, this interaction can lead to the creation of polymer-like structures (Figure 1) [47,48].

Understanding the interactions between sucrose and calcium carries significant technological implications, particularly for the sugar industry, where juice purification processes rely on the coagulating, neutralizing, and precipitating properties of calcium hydroxide (Ca(OH)_2_). In a typical process, this hydroxide is formed during the slaking of quicklime (CaO) in water, and its efficiency in sugar beet juice impurity removal is directly linked to the availability of reactive calcium species, represented by calcium oxide available and calcium oxide available in total lime. Since sucrose enhances the solubility of calcium oxide in aqueous environments, leading to an increased amount of available Ca^2+^ ions, it is strongly recommended that lime slaking in sugar factories be performed with sweet technological water. This approach could optimize juice purification, reduce lime consumption, and improve overall process efficiency—ultimately improving the final sugar quality. Further research into the mechanistic aspects of calcium–saccharate interactions could provide deeper insights into industrial sugar purification methods [49].

### 2.2. FT-NIR Spectra of the Milk of Lime Samples

**Figure 2:**
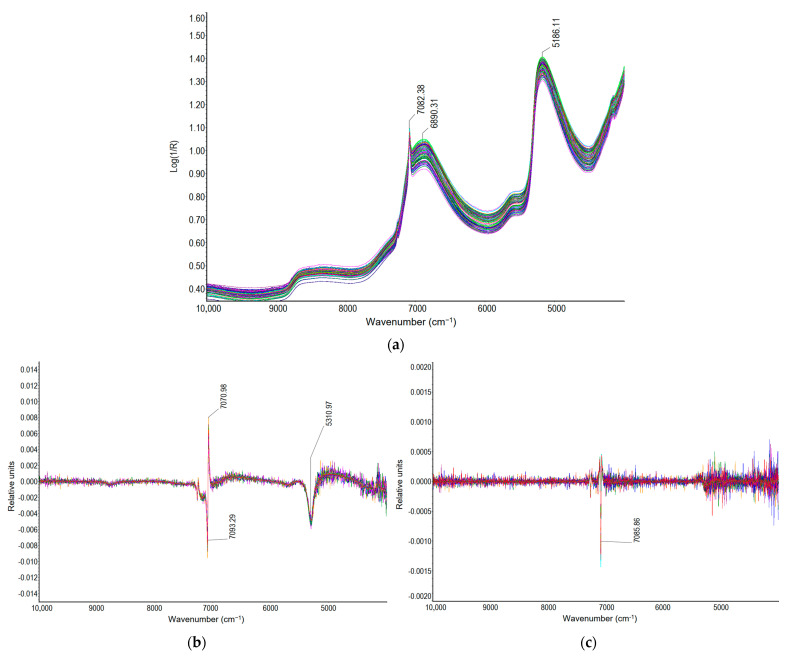
The FT-NIR spectra of the milk of lime (MOL) samples (with and without sucrose): (**a**) original FT-NIR spectra; (**b**) first derivative spectra; (**c**) second derivative spectra.

The original FT-NIR spectra of all MOL samples with and without sucrose and their first and second derivatives are shown in Figure 2. Milk of lime in all of the FT-NIR spectra exhibits several prominent absorption peaks, reflecting its chemical composition and characteristic molecular vibrations. By a visual inspection, the absorption peaks of different intensities are mainly around 7082 cm^−1^, 6890 cm^−1^, and 5186 cm^−1^. Two prominent peaks, at wavenumbers 7082 cm^−1^ and 5186 cm^−1^, indicate water molecules absorbed on the surface and the combination band of O-H stretching and bending, respectively. The 5186 cm^−1^ band is commonly associated with hydroxyl groups in calcium hydroxide and water, as well as sucrose [36,50], and is attributed to surface and interlayer hydroxyl groups of Ca(OH)_2_ [51]. The peak at 6890 cm^−1^ is mainly related to the first overtone of O-H stretching, while the O-H combination band is more accurately observed at 5186 cm^−1^ [27]. Additionally, it can be observed that all spectra exhibit similar shapes with significant spectral overlap between samples from the same dataset. Moreover, initial observations indicate that the spectra of all samples exhibit similar shapes and peaks.

The spectral similarities in the O-H overtone and combination regions result in substantial signal overlap, making it challenging to isolate the specific contributions of each compound. This spectral ambiguity is particularly evident in the 7000–6900 cm^−1^ region, where the O-H overtones of sucrose and calcium hydroxide exhibit nearly identical absorption features, obscuring the distinction between these two components (Figure 2a). While the application of the first and the second derivative transformations is intended to resolve such overlapping features by minimizing baseline drift and accentuating minor spectral differences, the inherent spectral overlap persists. As seen in the first derivative spectra (Figure 2b), the peaks at approximately 7082 cm^−1^ and 6890 cm^−1^ become more distinct yet remain strongly overlapping due to the shared OH functionalities in sucrose and calcium hydroxide. Similarly, in the second derivative spectra (Figure 2c), the peak at 5186 cm^−1^, corresponding to the O-H combination band, is further emphasized, yet it continues to reflect contributions from both components, making clear spectral differentiation difficult. Such spectral complexity is particularly problematic when attempting to develop predictive models for calcium oxide availability, total calcium oxide in lime, and sucrose content in milk of lime. Consequently, while the second derivative transformation provides some degree of spectral enhancement, the persistent overlap of OH bands necessitates the implementation of advanced chemometric techniques, such as PLS or PCR. These methods are essential to effectively deconvolute overlapping spectral features and to achieve accurate quantitative predictions in complex, multi-component mixtures like milk of lime.

### 2.3. Chemometric Analysis

**Figure 3:**
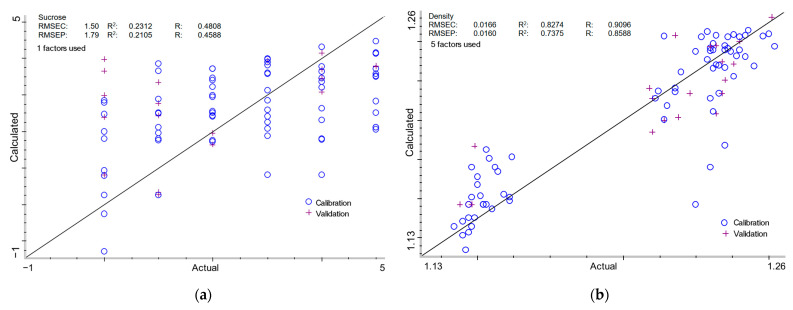

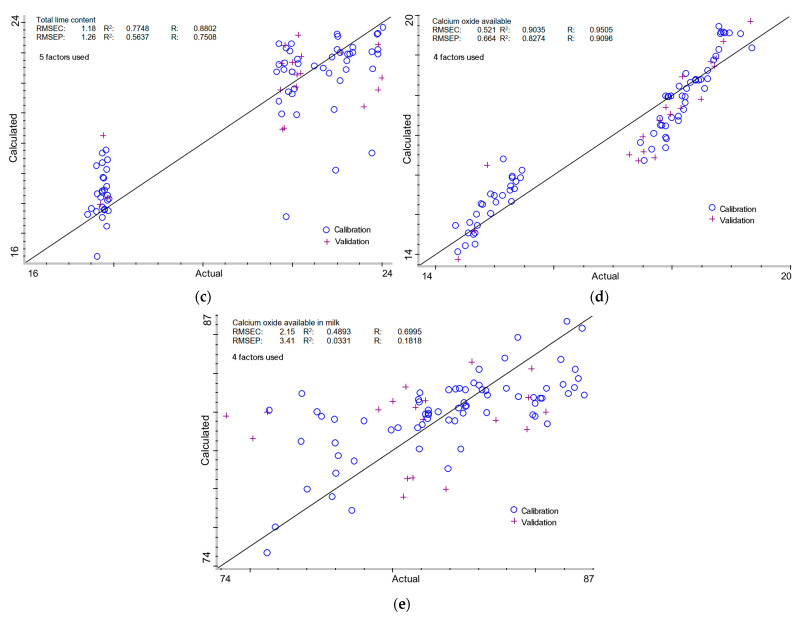
PLS calibration models for (**a**) sucrose content; (**b**) density; (**c**) total lime content; (**d**) calcium oxide available; and (**e**) % calcium oxide available in milk of lime.

**Figure 4:**
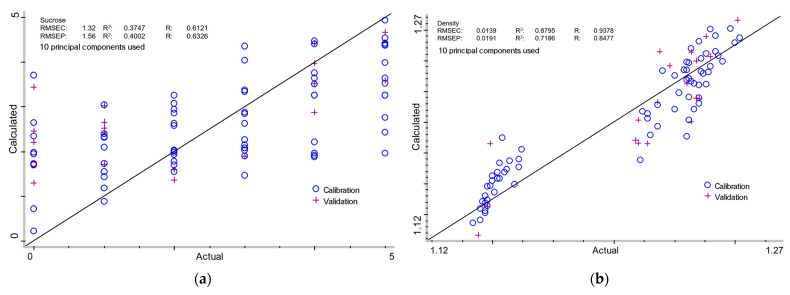

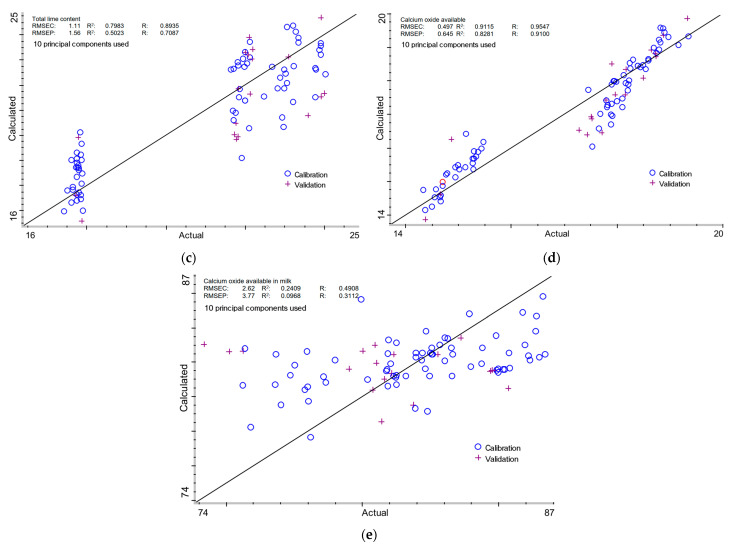
PCR calibration models for: (**a**) sucrose content; (**b**) density; (**c**) total lime content; (**d**) calcium oxide available; and (**e**) % calcium oxide available in milk of lime.

Quantitative determination of key parameters of milk of lime samples by Partial Least Squares (PLS) and Principal Component Regression (PCR) analyses is summarized in Table 2 and Table 3 and Figure 3 and Figure 4, respectively. The full spectral range was selected for both PLS and PCR analyses, as it produced more stable and reproducible results across all the tested parameters. Narrowing the spectral range often compromises model robustness, whereas using the entire spectrum preserves critical information, thereby enhancing predictive accuracy. The presented approach allows the models to be more adaptable to various types of samples and experimental conditions.

The spectral preprocessing methods listed in the tables for each analysis were selected to enhance model performance by addressing specific challenges associated with FT-NIR spectra, such as spectral overlap, noise, and baseline drift. The predictive performance of both models was evaluated based on calibration and validation errors, as well as determination coefficients (R^2^) for each parameter analyzed.

#### 2.3.1. Sucrose Content in Milk of Lime

The PLS model displayed a Root Mean Square Error of Calibration (RMSEC) of 1.50% and a validation error (RMSEP) of 1.79%, with corresponding determination coefficient (R^2^) of 0.2312 and 0.2105 (R: 0.4808 and 0.4588). The PCR model demonstrated significantly lower RMSEC (1.32%) and RMSEP (1.56%), coupled with marginally improved determination values: 0.3747 for calibration and 0.4002 for validation (R: 0.6121 and 0.6326). Despite the slight variation in predicted accuracy, the low R^2^ and R values in both models indicate that sucrose content remains problematic to assess using FTIR-based regression approaches due to chemical behavior in the alkaline milk of lime. Sucrose is known to form coordination complexes with calcium ions, particularly under alkaline conditions, which can affect both its solubility and spectroscopic signature. These calcium–saccharide complexes often produce minor spectrum alterations that are not immediately identifiable in multi-component systems [48]. In addition, sucrose can alter the crystallization and particle size distribution of calcium hydroxide during slaking, indirectly affecting the matrix in which it is embedded [52,53,54]. Changes in particle morphology or surface chemistry may introduce baseline fluctuations or matrix effects in FT-NIR spectra that degrade model stability and predictive accuracy for sucrose itself. Furthermore, unlike simple mono- or disaccharides, sucrose exhibits several overlapping O-H, C-O, and C-H combination bands, which can move or broaden in the presence of variable ionic strength and pH conditions characteristic of milk of lime [27,36,50,51,55]. Another restriction is the relatively small spectroscopic contribution of sucrose in this system to dominant absorbers like water and hydrated lime. Given that the sucrose concentrations investigated were often low (≤5% *w*/*w*), their spectral characteristics may fall near the noise level or be heavily veiled by adjacent stronger bands. This underlines the necessity for more sensitive feature extraction approaches to isolate sucrose-related changes.

Overall, the complexity of the milk of lime matrix, along with the unique behavior of sucrose in alkaline, calcium-rich conditions, poses a multifaceted obstacle to reliable FT-NIR-based quantification. Future studies may benefit from the inclusion of complementary spectroscopic approaches (e.g., Raman or mid-IR) or chemometric methodologies geared to extract low-intensity characteristics from overlapping profiles.

#### 2.3.2. Density of Milk of Lime

For density determination, PLS achieved RMSEC and RMSEP values of 0.0166 g/cm^3^ and 0.0160 g/cm^3^, respectively, with R^2^ values of 0.8274 and 0.7375 (R: 0.9096 and 0.8588). The PCR model demonstrated comparable performance, with RMSEC and RMSEP values of 0.0139 g/cm^3^ and 0.0191 g/cm^3^, respectively, and corresponding determination coefficients of 0.8795 (calibration) and 0.7186 (validation) (R: 0.9378 and 0.8477). The relatively high R^2^ values show both models are well-suited for density prediction, with a minor edge for PCR in calibration accuracy. However, the more stable validation performance of PLS suggests its suitability for practical applications. Nonetheless, to fully realize the predictive potential of these models, further studies involving a larger dataset are recommended. It is worth mentioning that the investigation of milk of lime density using FT-NIR represents a unique technique in this field. The use of FT-NIR spectroscopy for density investigation is well justified, as Fourier Transform Near-Infrared (FT-NIR) spectroscopy has been effectively employed to determine the density of various solutions and materials. Previous studies have demonstrated its efficiency in monitoring the density of linear low-density polyethylene (LLDPE) in both laboratory and industrial environments [56,57]. FT-NIR has also been used to predict the density of wood strands [58], solid wood [59], and *Eucalyptus grandis* [60]. In the petroleum industry, FT-NIR has shown potential for assessing the density of waxy crude oils [61]. The FT-NIR approach has been used to evaluate medicinal powders as well [62].

#### 2.3.3. Total Lime Content in Milk of Lime

The PLS model yielded RMSEC and RMSEP values of 1.18 and 1.26 g CaO/100 cm^3^, with R^2^ values of 0.7748 for calibration and 0.5637 for validation (R: 0.8802 and 0.7508). The PCR model demonstrated slightly better calibration performance (RMSEC = 1.11 g CaO/100 cm^3^, R^2^ = 0.7983, R = 0.8936) but lower validation accuracy (RMSEP = 1.56 g CaO/100 cm^3^, R^2^ = 0.5023, R = 0.7087). Both models exhibit reasonable predictive capabilities for total lime content, with PLS offering a more stable validation performance. The reduced accuracy of these models (lower R^2^ and R values) can be attributed not only to spectral interference induced by overlapping peaks of hydroxyl groups from sucrose and calcium hydroxide but also to the multiple calcium forms generated in the milk of lime, where calcium may be bonded in calcium oxide (CaO) or calcium hydroxide (Ca(OH)_2_), either in dissolved form or as a colloidal suspension [13,14]. Additionally, in highly alkaline solutions saturated with calcium ions, the predominant dissolved compound is Ca(OH)_2_(aq), further complicating spectral analysis. These different forms of calcium influence the spectral characteristics of the samples, making it more challenging to develop accurate predictive models.

#### 2.3.4. Calcium Oxide Available in Milk of Lime

In the PLS model, RMSEC and RMSEP values were equal to 0.521 and 0.664 g CaO/100 cm^3^, respectively, with a determination coefficient of 0.9035 for calibration and 0.8274 for validation (R: 0.9505 and 0.9096). Similarly, in the PCR model, Root Mean Square Errors were lower and RMSEC and RMSEP values were 0.497 and 0.645 g CaO/100 cm^3^, respectively, with corresponding R^2^ values of 0.9115 and 0.8281 (R: 0.9547 and 0.9100). Both models present reliable prediction capacities for the available calcium oxide measurement, and differences may be regarded as marginal. Nonetheless, similar to the density prediction models, these models demonstrate potential for calcium oxide estimation, but further refinement using a larger dataset would be advisable. Notably, during FT-NIR spectral analysis of the milk of lime samples, a distinctive and strong absorption band at approximately 7082–7083 cm^−1^ was consistently observed. This feature corresponds to the first overtone of the O-H stretching vibration in calcium hydroxide (Ca(OH)_2_) and has been verified in several prior investigations as a robust spectral marker for the presence of portlandite, particularly in cementitious systems [36,63]. According to the previous study [36], the second derivative of FT-NIR spectra revealed a strong linear relationship between the intensity at 7082 cm^−1^ and Ca(OH)_2_ content, with R^2^ values approaching 0.99 depending on the sample composition. Similarly, other research attributed bands at 7260 cm^−1^ and 7083 cm^−1^ to surface and interlayer hydroxyl groups in Ca(OH)_2_, respectively, and confirmed the disappearance of these peaks upon thermal dehydration—thereby validating their spectral assignments to hydrated lime phases [51]. This peak inherent in the samples tested in our study provides strong evidence that the calcium oxide available, as determined by wet chemical methods, is largely present in the hydrated state as Ca(OH)_2_. This spectral behavior substantiates the application of FT-NIR spectroscopy, particularly around the 7082 cm^−1^ wavelength, for the quantitative determination of available calcium oxide, as also demonstrated in studies on cement and concrete matrices [36,63]. Nevertheless, despite this promising correlation, additional challenges remain when analyzing more chemically complex systems such as lime–sucrose mixtures. Both calcium hydroxide and sucrose possess OH functional groups that give rise to overlapping absorption bands in the near-infrared region, which can complicate spectral interpretation and reduce model precision [47,48]. Furthermore, sucrose is known to interact with calcium ions, forming calcium–saccharate complexes that not only affect the physical state of Ca(OH)_2_ but also alter the vibrational characteristics of the system [45]. These interactions can result in modified peak shapes or shifts, especially in the critical 7000–7100 cm^−1^ range, potentially interfering with the quantitative signal for hydrated lime. Moreover, as indicated in recent work on sucrose–calcium interactions in sugar clarification systems, sucrose can influence the crystallinity and morphology of precipitated Ca(OH)_2_ [48,64]. These morphological changes can further impact FT-NIR signal intensity and reproducibility. As revealed in our findings and consistent with earlier literature, employing the 7082 cm^−1^ band as a calibration anchor remains a scientifically sound and potentially powerful strategy for improving the quantification of Ca(OH)_2_ in lime-based aqueous systems. However, to avail this potential, future modeling efforts should include enhanced preprocessing techniques, a broader calibration set, and attention to matrix-specific effects such as sucrose interference.

#### 2.3.5. Calcium Oxide Available in Total Lime of Milk of Lime

For the calcium oxide available in the total lime parameter, the PLS model yielded an RMSEC of 2.15% CaO and an RMSEP of 3.41% CaO, with corresponding R^2^ values of 0.4893 and 0.0331 (R: 0.6995 and 0.1818). This indicates significantly lower predictive accuracy compared to other parameters analyzed. The PCR model showed an even poorer performance, with an RMSEC of 2.62% CaO and an RMSEP of 3.77% CaO, and lower determination and correlation coefficients (R^2^ value of 0.2409 for calibration and 0.0968 for validation; R: 0.4908 and 0.3112). These results illustrate the limited capability of both models to accurately predict the percentage of calcium oxide available in total lime, despite their relatively strong performance in predicting the absolute amount of calcium oxide available (g CaO/100 cm^3^). This discrepancy can be partially explained by the fact that the percentage value is calculated as the ratio of calcium oxide available to total lime content and is thus more sensitive to error propagation. As such, any error in either of the two underlying variables—especially in total lime content, for which both models (PLS and PCR) showed only moderate predictive accuracy—can be amplified when calculating their ratio. This amplification may introduce noise, which refers to random variability or fluctuations in the predicted signal that are not related to actual chemical changes in the sample. As a result, the increased noise in the ratio-based calculation leads to reduced robustness and reliability in the %CaO predictions. Furthermore, total lime content itself is influenced by the heterogeneous distribution of different calcium compounds: CaO, Ca(OH)_2_, and sucrose–calcium complex forms, which may exist in distinct physical states (dissolved, suspended, or colloidal) and respond differently to FT-NIR radiation. As described previously, overlapping O-H absorption bands from Ca(OH)_2_ and sucrose contribute to spectral congestion, complicating individual signal attribution and increasing the likelihood of model overfitting or underfitting in ratio-based parameters. The particularly low determination coefficient R^2^ of 0.0331 for the PLS model underscores the extremely weak predictive relationship observed for the calcium oxide percentage in total lime. This value further emphasizes the limitations of the model in effectively capturing the variability in % CaO content, likely due to the inherent propagation of error from the ratio calculation. As noted previously, combining two independently predicted parameters (available CaO and total lime content) into a single ratio variable can magnify noise and obscure genuine spectral features, particularly in the presence of overlapping OH absorption bands. Consequently, the PLS model for % CaO should be interpreted with caution, as the observed R^2^ value indicates minimal predictive capability and a high likelihood of overfitting or underfitting. Moreover, the weak correlation observed in this case may also reflect the extension of uncertainty from two independently predicted quantities. While the prediction of available CaO alone yielded strong R^2^ values (>0.90), and total lime showed moderate performance, combining both into a single ratio variable magnifies the effect of even small inconsistencies, especially in validation. Lastly, since sucrose not only overlaps spectrally with Ca(OH)_2_ but also modifies the precipitation behavior of CaO and Ca(OH)_2_ and their solubility, it may indirectly distort the ratio between available and total lime content in a non-linear and sample-dependent manner. This introduces additional complexity that traditional linear regression models, such as PLS and PCR, may struggle to resolve without non-linear enhancements or variable selection.

In summary, although FT-NIR spectroscopy demonstrated a high potential for quantifying available CaO content in absolute terms, its predictive power diminishes when applied to relative parameters like %CaO due to error propagation, spectral overlap, and the compounded effect of matrix complexity. The evaluation of model performance was primarily based on the coefficient of determination (R^2^), as it provides a more robust assessment of predictive strength by accounting for the proportion of variance explained by the model. Detailed evaluation of R^2^ values reveals that the models for sucrose content (PLS: R^2^ 0.2312; PCR: R^2^ 0.3747) and %CaO (PLS: R^2^ 0.4893; PCR: R^2^ 0.2409) exhibit limited predictive power. Conversely, the models for density and available calcium oxide achieved R^2^ values exceeding 0.80, indicating strong predictive capability.

## 3. Materials and Methods

### 3.1. Milk of Lime Preparation

Quicklime (CaO) used for the preparation of milk of lime was obtained from the calcination of limestone (CaCO_3_). The quicklime was sourced from a Polish sugar factory directly after the calcination process (1):

CaCO_3_ (solid) + 178 kJ → CaO (solid) + CO_2_ (1)

The collected quicklime particles were carefully crushed to a uniform size range of 10 to 20 mm to ensure a consistency sample, using an agate mortar (C. Giese GmbH & Co. KG, Barntrup, Federal Republic of Germany) to minimize contamination and preserve the chemical integrity of the material. Then, the crushed material was mixed to further homogenize the batch. Subsequently, precisely measured amounts of quicklime were added to a controlled quantity of water or a water–sucrose solution, preheated to a temperature of 60 °C (2):

CaO (solid) + H_2_O → Ca(OH)_2_ (milk of lime) + 65 kJ (2)

The slaking process was carried out in a custom-designed setup consisting of a sealed and thermally insulated vessel equipped with a mechanical stirrer, ensuring uniform mixing and minimizing heat loss during the reaction (the components used were manufactured by Heidolph Instruments GmbH & Co. KG, Schwabach, Federal Republic of Germany). The duration of the slaking process was set at 20 min, during which the mixture was kept under controlled conditions to ensure complete hydration. The resulting milk of lime was cooled to a final temperature of 20 °C to stabilize the suspension and prevent undesirable alterations in the physical properties of the hydrated lime. The mass proportions of individual components used in the milk of lime preparation are presented in Table 4.

### 3.2. Chemical and Physicochemical Analysis

Milk of lime density was measured with the hydrometers (Carl Roth GmbH + Co. KG, Karlsruhe, Federal Republic of Germany, measuring range: 1.100–1.200 g/cm^3^ and Amarell H801062 Precision GmbH, Mannheim, Federal Republic of Germany, measuring range: 1.200–1.300 g/cm^3^, both with measurement accuracy 0.001 g/cm^3^) in accordance with the Polish standard BN-80 5537-03 [65]. Measurements were taken at 20 °C, as per the standard’s requirements for accurate density determination.

Total lime content and calcium oxide available were measured by using the titration methods described in European Norm EN 459-2 [66]. Total lime content refers to the sum of all lime components present in a sample, encompassing both active lime (free lime, CaO—unreacted calcium oxide) and slaked lime (Ca(OH)_2_—calcium hydroxide formed during the slaking process). Mathematically, it can be expressed as (3):

Total Lime Content = CaO + Ca(OH)_2_ (3)

Available calcium oxide (CaO Available) refers to the amount of reactive calcium oxide present in a lime-based suspension, such as milk of lime. It represents the portion of calcium oxide that is capable of reacting with water or other compounds under specified conditions, and it is typically expressed as (4):

CaO Available = CaO − CaO bound as Ca(OH)_2_ (4)

Calcium oxide available in total lime (in %) refers to the quantity of reactive calcium oxide (CaO) present in the milk of lime suspension that is capable of reacting under specific conditions. It represents the fraction of calcium oxide that remains unreacted after the slaking process and is available for further chemical reactions, expressed as (5):

CaO Available in total lime = (CaO Available/CaO total) × 100%(5)

The sucrose concentration was determined based on the precise weights of the components used during its preparation, as presented in Table 4.

### 3.3. Near-Infrared Spectroscopy

All the spectra were collected using the Thermo ScientificTM NicoletTM iS50 FT-IR Spectrometer (Thermo Fisher Scientific Inc., Waltham, MA, USA). NIR spectra were recorded using OMNIC 9 Software ver. 9.3 (Thermo Fisher Scientific Inc., Waltham, MA, USA). The spectrometer was equipped with a Thermo Scientific™ Integration Sphere connected to a Thermo Scientific™ Sample Cup Spinner. This combination simplified the collection of diffuse reflectance spectra for bulk, heterogeneous samples. The Sample Cup Spinner, combined with the Integrating Sphere, collected spectra, which were representative of heterogeneous samples, thus eliminating the need to take multiple spectra of the same sample. Using the integrating sphere diffuse reflectance sampling module, approximately 15 g of milk of lime was loaded into a sample cup rotator with an inner diameter of 4.78 cm. The spectra were recorded in the wavenumber range of 10,000–4000 cm^−1^ with a resolution of 8.00 cm^−1^. Each FT-NIR spectrum was obtained as the average of 32 scans of the sample. The room temperature was 20 °C. A total of 90 spectra were obtained by analyzing 18 distinct types of milk of lime, each measured in five replicates to ensure reliability and reproducibility. The replicate measurements were performed not only to verify the consistency of the FT-NIR spectral data but also as part of simultaneous physicochemical and chemical analyses, during which FT-NIR spectra were additionally collected to maintain comprehensive data alignment. The diffuse reflectance signal of the NIR spectrum was expressed as log (1/R) (R = reflectance). The software used for data manipulation and chemometric analysis was TQ Analyst 9 Software (Thermo Electron Corporation, Waltham, MA, USA). In order to develop the models, 80% of the samples (72) were taken in the calibration group and 20% of the samples (18) in the external validation (cross-validation). The samples were placed in the two groups at random.

### 3.4. Chemometric Techniques

Since the application of the FT-NIR technique was considered with the aim of developing an optimal yet straightforward model, Partial Least Squares (PLS) analysis was chosen, and Principal Component Regression (PCR) analysis was also used for comparison. While both methods address multicollinearity and dimensionality reduction, PLS offers a key advantage by explicitly modeling the relationship between spectral data and the target variables during model construction. In contrast to PCR, which calculates principal components without addressing the dependent variables, PLS takes latent variables that maximize correlation with the response variables, resulting in enhanced prediction accuracy. Moreover, PLS displays superior reliability in managing extremely collinear spectral data, noise, and irrelevant variance—common issues in FTIR spectroscopy. These properties establish PLS as a more trustworthy approach for assessing factors such as density, sucrose content, and calcium in milk of lime [67,68,69,70]. PLS is a developed generalization of multiple linear regression (MLR), but unlike MLR, it can analyze data with strongly collinear (correlated), noisy, and numerous X-variables and also simultaneously model several response variables Y [71,72]. To minimize the undesired contributions to the NIR signals, various spectral pretreatments were used together with derivatives and smoothing. Chemometric models were constructed using raw data and following preprocessing of the spectra, which included Multiplicative Scatter Correction (MSC), standard normal variate (SNV), Savitzky-Golay filter, Norris derivative filter, mean centering, baseline correction, smoothing, and combinations of these [73,74]. Although various spectral preprocessing methods (including SNV, SGF, NDF, and MPBC) were initially tested during model development, only those techniques that yielded the most reliable and statistically robust results were retained in the final PLS and PCR models. Therefore, only these selected methods are presented in Table 2 and Table 3 to avoid unnecessary complexity. The accuracy and reliability of models were assessed by using statistical error metrics, including the Root Mean Square Error of Calibration (RMSEC) and the Root Mean Square Error of Prediction (RMSEP) [75]. RMSEC evaluates how well the model matches the calibration dataset. It is determined as the Root Mean Square of the differences between the actual reference values and the values predicted by the model for the calibration samples. RMSEC is calculated according to Equation (6).

(6) $$RMSEC=\sqrt{\frac{1}{n}\sum_{i=1}^{n}{\left(y_{i}-\hat{y_{i}}\right)}^{2}}$$

where ${(y}_{i})$ represents the actual reference value for the calibration sample, $\hat{{(y}_{i})}$ is the predicted value from the calibration model, and (n) is the number of calibration samples. A lower RMSEC value indicates a better fit of the model to the calibration data. RMSEP assesses the prediction performance of the model using an independent validation or test dataset. It is computed according to Equation (7), similar to RMSEC, but is applied to new, unseen samples.

(7) $$RMSEP=\sqrt{\frac{1}{m}\sum_{i=1}^{m}{\left(y_{i}-\hat{y_{i}}\right)}^{2}}$$

where ${(y}_{i})$ represents the actual reference value for the validation sample, $\hat{{(y}_{i})}$ is the predicted value from the validation model, and (m) is the number of validation samples. A lower RMSEP value indicates high predictive accuracy, and the difference between RMSEC and RMSEP gives insight into model resilience. A significant increase in RMSEP compared to RMSEC indicates overfitting, suggesting that the model is overly fitted to the calibration data and may not generalize well to new samples. In this study, the Spectrum Outlier Diagnostic (SOD) was excluded due to the uniform and repeatable sample preparation technique, as well as the stable measurement conditions. All samples were prepared using the same standardized process, minimizing the potential of artifacts due to sample heterogeneity or contamination. Furthermore, the measurement settings were carefully controlled, with temperature and other external parameters kept consistent to ensure the reliability and consistency of the spectrum data. A comprehensive evaluation of the collected FTIR spectra indicated no dramatic differences amongst the samples, with all spectra displaying consistent peak positions and intensities, suggesting the absence of outlier data. No unexpected discrepancies or anomalies were identified in the spectrum data, verifying the absence of erroneous readings. Additionally, the FTIR instrument was well-maintained, calibrated, and functioned effectively during the analysis. Given the consistent operating conditions and the absence of any instrument-related difficulties, the exclusion of the Spectrum Outlier Diagnostic was regarded as reasonable, and the results obtained were considered reliable and representative of the examined samples.

The following criteria were established for model evaluation: both the Root Mean Square Error of Calibration (RMSEC) and Root Mean Square Error of Prediction (RMSEP) were required to be as low as possible, ideally below 5% of the data range. This threshold ensures that the model functions with high accuracy for both the calibration set and for predicting new, unseen data. The coefficient of determination (R^2^) is considered the primary indicator of model quality, as it quantifies the proportion of variance in the reference values that is captured by the predictive model. Given its clear interpretative power, R^2^ provides a more robust assessment of predictive capability than the correlation coefficient (R), particularly in quantitative regression contexts. The correlation coefficient (R) is retained alongside R^2^ as it directly conveys the strength of the linear relationship between observed and predicted values. For model evaluation, the following criteria were applied: models with R^2^ values above 0.95 are considered very strong predictors, indicating excellent quantitative agreement; values between 0.90 and 0.95 suggest strong predictive capacity, while R^2^ values below 0.90 but above 0.80 are categorized as moderate predictors. Models with R^2^ values below 0.80 are regarded as having limited predictive potential and may require further refinement through additional data or model adjustments. Additionally, to minimize the risk of overfitting and to ensure that the models generalize well to new data, RMSEC and RMSEP values were monitored for similarity. This practice serves as an additional check for model robustness, reinforcing the overall reliability of the presented regression models.

### 3.5. Statistical Analysis

The results of all independent experiments were expressed as the mean ± standard deviation (SD). The Statistica 13.0 software (StatSoft, Inc., Tulsa, OK, USA) was used for the statistical data analyses. The significant differences were estimated by one-way analysis of variance (ANOVA) followed by Tukey’s Honest Significant Difference (HSD) test. Differences were considered to be significant when *p*-values were less than 0.05 (*p* < 0.05).

## 4. Conclusions

The study explored the use of Fourier Transform Near-Infrared (FT-NIR) spectroscopy combined with chemometric modeling techniques like PLS and PCR to analyze milk of lime (MOL) used in the sugar industry. This approach offers a rapid, non-invasive, and sustainable alternative for assessing MOL composition.

The availability of calcium oxide, as well as its proportion in the total lime, was found to be sensitive to the presence of sucrose. Sucrose not only enhanced CaO solubility and increased the amount of Ca(OH)_2_ formed but also probably influenced the interaction between lime particles and water, leading to the formation of calcium–saccharate complexes, which affected the physicochemical properties of the suspension and may have notable implications for sugar beet or cane juice clarification processes.

A consistent spectral band at approximately 7082–7083 cm^−1^, associated with group O-H in Ca(OH)_2_, confirms the utility of this peak as a spectral marker for reactive lime species, particularly in complex aqueous matrices. However, the overlap of O-H bands from sucrose and calcium hydroxide introduced significant challenges in accurately modeling certain parameters, like sucrose content and the percentage of calcium oxide in the lime.

Chemometric analysis with PLS and PCR provided strong predictions for density and calcium oxide availability, but the results for sucrose content and lime ratios were moderate to weak. The predictive models for sucrose content, particularly PCR, demonstrated poor performance, as evidenced by the low R^2^ values (0.21 for PLS and 0.40 for PCR). The similarity between calibration and validation plots (Figure 3a and Figure 4a) suggests potential overfitting and a lack of generalization capability, likely due to the substantial spectral overlap between sucrose and calcium hydroxide. Further model optimization and alternative preprocessing strategies may be necessary to address these limitations. This disparity reflects the combined influence of overlapping spectral features, propagation of prediction errors in ratio-based parameters, and variability in calcium speciation. Still, the accurate prediction of density indicates that FT-NIR can be a valuable tool for monitoring milk of lime in real-time without damaging samples, supporting green chemistry and process optimization efforts.

While the predictive models for density and available calcium oxide achieved robust performance metrics (R^2^ > 0.80), the models for sucrose content and %CaO exhibited significantly lower R^2^ values, reflecting inherent spectral overlap and matrix complexity in the milk of lime samples. The emphasis on R^2^ as the primary indicator of predictive strength provides a more accurate representation of model quality, with implications for future studies aiming to enhance the calibration strategy, particularly for complex matrices with overlapping spectral features.

A notable observation in this study was the potential presence of underlying group structures in the dataset, particularly for density, total lime content, and calcium oxide available. Although all samples originated from a single, well-mixed batch of quicklime, subtle sources of variability may have contributed to apparent grouping effects. Developing comprehensive and robust predictive models requires a sufficiently large dataset to fully capture such variability. Nevertheless, the current results demonstrate that FT-NIR spectroscopy is a promising tool for monitoring milk of lime composition, providing rapid, non-invasive assessments that align well with industrial requirements.

In conclusion, FT-NIR spectroscopy, when applied with appropriate preprocessing and calibration strategies, offers a rapid and environmentally sustainable method for assessing critical quality attributes of milk of lime. Its versatility, reagent-free operation, and non-invasive nature make FT-NIR well-suited for industrial applications. Future studies should aim to deepen the understanding of sucrose–calcium interactions, improve spectral interpretation techniques, and develop advanced modeling approaches to enhance accuracy in parameters such as %CaO. Strengthening chemometric strategies for complex matrices could unlock broader applications in sugar processing and other industries.

## Figures and Tables

**Table 1:** Physicochemical analysis of milk of lime (MOL).

CaO:H_2_0 or Sucrose Solution	Sucrose (%)	Density (g/cm^3^)	Total Lime Content (g CaO/100 cm^3^)	Calcium Oxide Availability (g CaO/100 cm^3^)	Calcium Oxide Availability in Total Lime (%)	Change in Calcium Oxide Availability Relative to Water Solution (Δ%)
	0	1.145 ± 0.002 ^a^	17.79 ± 0.19 ^a^	14.53 ± 0.17 ^a^	81.67 ± 0.88 ^a^	0.00
	1	1.148 ± 0.001 ^a^	17.78 ± 0.09 ^a^	14.61 ± 0.18 ^ab^	82.15 ± 0.53 ^ab^	0.48
	2	1.148 ± 0.001 ^a^	17.67 ± 0.11 ^a^	14.74 ± 0.17 ^abc^	83.39 ± 0.61 ^bc^	1.72
1:6	3	1.151 ± 0.002 ^b^	17.80 ± 0.02 ^a^	14.98 ± 0.11 ^bc^	84.17 ± 0.48 ^cd^	2.50
	4	1.154 ± 0.002 ^b^	17.78 ± 0.05 ^a^	15.28 ± 0.09 ^c^	85.91 ± 0.29 ^de^	4.24
	5	1.160 ± 0.001 ^c^	17.76 ± 0.11 ^a^	15.36 ± 0.08 ^d^	86.53 ± 0.15 ^e^	4.86
	0	1.211 ± 0.002 ^a^	21.91 ± 0.15 ^a^	17.59 ± 0.26 ^a^	80.28 ± 0.64 ^a^	0.00
	1	1.215 ± 0.004 ^b^	21.93 ± 0.20 ^a^	17.72 ± 0.15 ^ab^	80.81 ± 0.33 ^b^	0.52
	2	1.219 ± 0.003 ^c^	21.94 ± 0.23 ^a^	18.04 ± 0.12 ^b^	82.23 ± 0.28 ^c^	1.94
1:5	3	1.227 ± 0.002 ^d^	21.92 ± 0.09 ^a^	18.18 ± 0.13 ^b^	82.93 ± 0.34 ^d^	2.64
	4	1.232 ± 0.002 ^de^	21.94 ± 0.19 ^a^	18.62 ± 0.17 ^c^	84.86 ± 0.11 ^e^	4.57
	5	1.233 ± 0.003 ^e^	21.92 ± 0.16 ^a^	18.68 ± 0.16 ^c^	85.24 ± 0.18 ^ef^	4.96
	0	1.232 ± 0.002 ^a^	23.51 ± 0.45 ^a^	17.81 ± 0.26 ^a^	75.76 ± 1.51 ^a^	0.00
	1	1.233 ± 0.002 ^a^	23.44 ± 0.41 ^a^	17.91 ± 0.26 ^a^	76.39 ± 0.63 ^ab^	0.63
	2	1.233 ± 0.003 ^a^	23.27 ± 0.43 ^a^	18.10 ± 0.26 ^a^	77.75 ± 0.30 ^b^	1.99
1:4	3	1.239 ± 0.003 ^b^	23.35 ± 0.58 ^a^	18.46 ± 0.26 ^b^	79.05 ± 1.05 ^c^	3.29
	4	1.241 ± 0.002 ^bc^	23.28 ± 0.39 ^a^	18.84 ± 0.26 ^c^	80.94 ± 0.40 ^d^	5.18
	5	1.249 ± 0.003 ^a^	23.39 ± 0.52 ^a^	19.02 ± 0.26 ^d^	81.30 ± 0.47 ^d^	5.54

^a–f^ Different letters in the same column of any group indicate statistically significant differences; analysis of variance (ANOVA); post hoc: Tukey HSD test; *p* < 0.05.

**Table 2:** PLS quantitative analysis of milk of lime, performed using TQ Analyst Software ver 9.4.

Data Format			MSC	PLSf	Calibration			Validation		
RS	1st	2nd			RMSEC	R^2^	R	RMSEP	R^2^	R
Sucrose content (%)										
-	+	-	+	1	1.50	0.2312	0.4808	1.79	0.2105	0.4588
Density (g/cm^3^)										
-	+	-	+	5	0.0166	0.8274	0.9096	0.0160	0.7375	0.8588
Total lime content (g CaO/100 cm^3^)										
-	+	-	-	5	1.18	0.7748	0.8802	1.26	0.5637	0.7508
Calcium oxide available (g CaO/100 cm^3^)										
+	-	-	-	4	0.521	0.9035	0.9505	0.664	0.8274	0.9096
% Calcium oxide available (% CaO)										
-	-	+	-	4	2.15	0.4893	0.6995	3.41	0.0331	0.1818

RS: raw spectra; 1st: first derivative; 2nd: second derivative; MSC: Multiplicative Scatter Correction; PLSf: number of PLS factors; RMSEC: Root Mean Square Error of Calibration; RMSEP: Root Mean Square Error of Prediction; R^2^: determination coefficient; R: correlation coefficient. Other TQ method parameters selected for all PLS analysis variants: spectral range: 10,000–4000 cm^−1^; Data Normalization: mean centering technique; Fit Value Algorithm: simple (measured from zero); concentration weighted PLS: weight by component concentration values for each standard; PLS factors: always use one-a-time cross-validation and automatic update.

**Table 3:** PCR quantitative analysis of milk of lime, performed using TQ Analyst Software ver. 9.4.

Data Format	MSC	PCU	Calibration			Validation		
RS			RMSEC	R^2^	R	RMSEP	R^2^	R
Sucrose content (%)								
+	-	10	1.32	0.3747	0.6121	1.56	0.4002	0.6326
Density (g/cm^3^)								
+	-	10	0.0139	0.8795	0.9378	0.0191	0.7186	0.8477
Total lime content (g CaO/100 cm^3^)								
+	-	10	1.11	0.7983	0.8935	1.56	0.5023	0.7087
Calcium oxide available (g CaO/100 cm^3^)								
+	-	10	0.497	0.9115	0.9547	0.645	0.8281	0.9100
% Calcium oxide available (% CaO)								
+	+	10	2.62	0.2409	0.4908	3.77	0.0968	0.3112

RS: Raw spectra; MSC: Multiplicative Scatter Correction; PCU: Principal Components; RMSEC: Root Mean Square Error of Calibration; RMSEP: Root Mean Square Error of Prediction; R^2^: determination coefficient; R: correlation coefficient. Other TQ method parameters selected for all PCR analysis variants: Spectral range: 10,000–4000 cm^−1^; Data Normalization: mean centering technique; Fit Value Algorithm: Simple (measured from zero).

**Table 4:** Milk of lime (MOL) composition with different CaO and water or sucrose solutions ratios.

CaO:Water or Sucrose Solution	CaO Mass (g)	H_2_O Mass (g)	Sucrose Mass (g)	Sucrose Concentration (% *w*/*w*)	Total Mass (g)
1:4	100	400	0	0	500
	100	395	5	1	500
	100	390	10	2	500
	100	385	15	3	500
	100	380	20	4	500
	100	375	25	5	500
1:5	100	500	0	0	600
	100	494	6	1	600
	100	488	12	2	600
	100	482	18	3	600
	100	476	24	4	600
	100	470	30	5	600
1:6	100	700	0	0	700
	100	700	7	1	700
	100	700	14	2	700
	100	700	21	3	700
	100	700	28	4	700
	100	700	35	5	700

## Data Availability

The data presented in this study are available on request from the corresponding author. The data are not publicly available because they are part of the authors’ own research.
